# Wilms’ tumor 1 (WT1) as a prognosis factor in gynecological cancers: A meta-analysis

**DOI:** 10.1097/MD.0000000000011485

**Published:** 2018-07-13

**Authors:** Jingjing Lu, Yang Gu, Qing Li, Huanxin Zhong, Xiaoxue Wang, Zhenxia Zheng, Wenfeng Hu, Lanling Wen

**Affiliations:** a Department of Gynecology and Obstetrics, Zhongshan Hospital Affiliated to Xiamen University; b Department of Orthopedics, First Affiliated Hospital of Xiamen University; c Department of Gynecology and Obstetrics, Hong’ai Hospital, Xiamen, Fujian; d Department of Gynecology and Obstetrics, Quzhou People's Hospital, Quzhou, Zhejiang, China.

**Keywords:** gynecological cancer, meta-analysis, prognosis, WT1

## Abstract

The oncogenic role of Wilms’ tumor 1 (WT1) which is regarded as a promising target antigen for cancer immunotherapy has been demonstrated in many types of cancer, but the relationship between expression of WT1 and the prognosis value in gynecological cancer reminds unclear.

We performed a meta-analysis with thirteen published studies including 2205 patients searched from PubMed, EMBASE, Web of Science, and Google Scholar, whose results are expressed by overall survival (OS) or disease-specific survival (DSS) or disease-free survival or relapse/recurrence-free survival (RFS) or progression-free survival (PFS) in patients with gynecological cancer. The hazard ratio (HR) with its 95% confidence interval (CI) were calculated to investigate prognostic of WT1 expression in patients with gynecological cancer.

Finally, the overexpression of WT1 was borderlinely associated with poor OS (metaHR = 1.51, 95% CI = 0.98–2.31) in univariate model. We found a significant association with poor DSS (metaHR = 1.61, 95% CI = 1.24–2.08) and DFS/RFS/PFS (metaHR = 2.06, 95% CI = 1.22–3.46). The subgroup analyses revealed that the expression of WT1 predicted the poor DSS (metaHR = 1.82, 95% CI = 1.42–2.73), and DFS/RFS/PFS (metaHR = 2.51, 95% CI = 1.81–3.48) in patients with ovarian cancer. In summary, WT1 overexpression indicates a poor prognosis in patients with some gynecological tumors, but more studies are needed to confirm these findings.

## 1 Introduction

Cervical cancer, endometrial cancer (EC), ovarian cancer (OC), vulvar cancer, vaginal cancer, uterine sarcoma (US), and gestational trophoblastic cancer are included as gynecological cancers according to the division of *Williams Gynecology* 3rd edition. The 5-year survival statistics of the gynecological cancers are quite poor despite well-established surgical and chemotherapeutic treatments. For example, more than 70% of OC patients are diagnosed with late-stage due to lacking of specific initial symptoms. Late-stage patients’ 5-year overall survival (OS) is less than 20%, while the data show that it can reach approximately 90% among early-stage disease patients.^[1]^ That is the reason why we use specific molecular markers as an important prognostic factor to monitor gynecological cancer for either therapeutic effect or follow-up purpose.

The Wilms’ tumor 1 (WT1), located at chromosome 11p13, was identified as a gene responsible for the development of Wilms’ tumor at first.^[2]^ During last decades, WT1 has been identified as a contributor to carcinogenesis in various kinds of human cancers including leukemia and myelodysplastic syndromes, brain cancer, neuroblastoma, lung cancer, breast cancer, head and neck squamous cell carcinoma, thyroid cancer, esophageal cancer, renal cell carcinoma as well as in gynecological tumor such as OC, EC, and US.^[3–7]^

Although the prognostic and immunotherapeutic role of WT1 has been demonstrated in a variety of nongynecological cancer types,^[8,9]^ the prognostic value of WT1 expression in gynecological tumor still remains unclear. We evaluated the prognostic value of WT1 in gynecological cancers through meta-analysis to elucidate its potential use in practice.

## 2 Materials and methods

This meta-analysis was performed according to the statement for reporting systematic reviews and meta-analyses.^[10]^ Previously published studies were summarized and analyzed in this study (ethics approval was unnecessary).

### 2.1 Search strategy

A thorough search of PubMed, EMBASE, Web of Science, and Google Scholar was conducted to retrieve studies measuring WT1 expression and survival of patients with gynecological cancers from 2000 to August 2017. The search terms included (“WT1” or “Wilms’ tumor 1” or “Wilms’ tumor gene 1” or “Wilms’ tumor protein 1”) and (“gynecological” or “ovarian” or “cervical” or “endometrial” or “vulvar” or “vaginal” “or “uterine” or “gestational trophoblastic”) and (“cancer” or “tumor” or “malignancy” or “carcinoma or sarcoma” and “prognosis or survival”). The language was limited to English only. Results were restricted to human studies of gynecological cancer and 363 entries were found totally.

### 2.2 Study eligibility

Inclusion criteria contained an evaluation of overexpression of WT1 linked to OS, disease-specific survival (DSS), disease-free survival (DFS), progression-free survival (PFS), and recurrence-free survival (RFS). WT1 expression was evaluated by antigen-based or mRNA-based method. Reviews, clinical endpoints other than OS/DSS/DFS/RFS/PFS, studies that enrolled less than 50 patients, and studies without data that could be used for calculating hazard ratio (HR) with its 95% confidence interval (95% CI) were excluded. In case of multiple publications from the same institution, the most informative report was included. Any disagreement was resolved by discussion among all investigators until a final consensus was reached.

### 2.3 Data extraction

Two investigators extracted data independently and disagreements were worked out through discussion. Data retrieved from the studies included the following: author, country, year of publication, cancer type, recruitment time, follow-up time, OS/DSS/DFS/RFS/PFS, cut-off value of positive/negative WT1 expression (Table 1), univariate or multivariate HR and 95% CI estimation. We preferred multivariate HRs if both were available for studies because intermixed factors were included in the multivariate analyses. Some HRs were extracted from the tables or Kaplan–Meier curves for both WT1 positive and negative expression groups.^[11]^

**Table 1:**
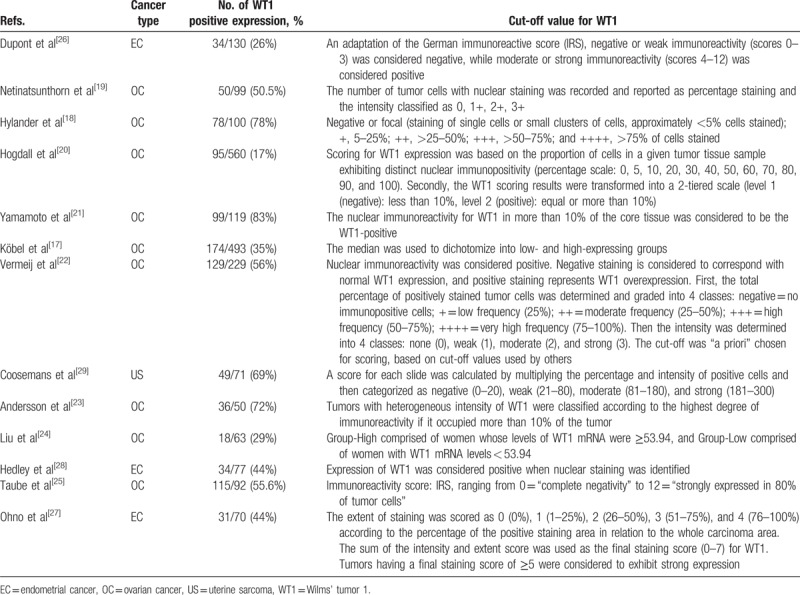
Evaluation the cut-off value for Wilms’ tumor 1 (WT1) in the selected studies.

### 2.4 Quality assessment

Quality assessment for cohort studies in our meta-analysis was assessed using the Newcastle-Ottawa Non-Randomized scale (NOS) as recommended by the Cochrane Non-Randomized Studies Methods Working Group^[12,13]^ The judgment was on 3 board perspectives: study group's selection (4 criteria), study group's comparability (1 criteria), and ascertainment of outcome of interested (3 criteria). NOS ranges from 0 to 9 scores, proving studies showing a score ≥ 5 is considered as methodologically high quality. A consensus of NOS score for each study was achieved by discussion of all investigators (Table 2).

**Table 2:**
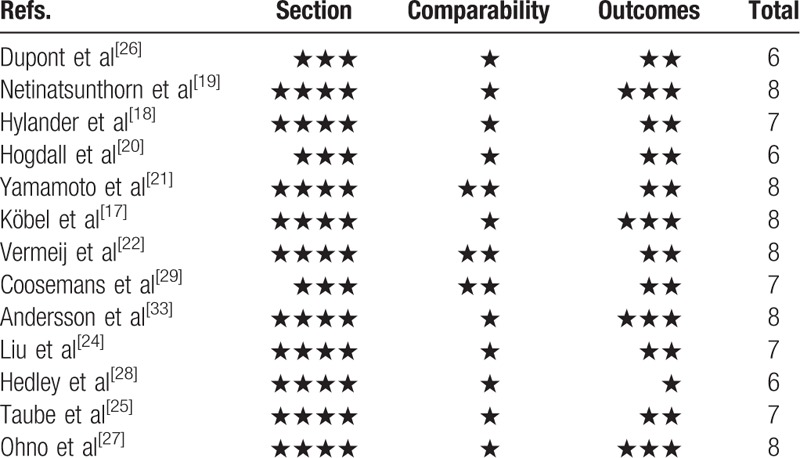
Newcastle-Ottawa Scale scores for nonrandomized studies.

### 2.5 Statistical analysis

All the data were analyzed with the RevMan 5.3 analysis software (Cochrane Collaboration, Oxford, UK). The HRs with their corresponding 95% CI estimates were calculated and pooled to assess the association of WT1 overexpression with OS/DSS/DFS/RFS/PFS. An HR > 1 indicated a worse prognosis in patients with WT1 overexpression. Two models of meta-analysis were used, including the random-effects model and the fixed-effects model, conducted respectively by Mantel's and DerSimonian's methods.^[14,15]^ Statistical intrastudy heterogeneity was evaluated by the *I*^2^ value to quantify the proportion of the total variation. The *I*^2^ values of 25%, 50%, and 75% were the cutoff points of low, moderate, and high heterogeneity, respectively. A subgroup analysis depending on the characteristics of gynecological cancers was conducted to explore possible explanations for heterogeneity if high heterogeneity existed.^[16]^ Fixed-effects model was used to pool the results if relatively low or moderated heterogeneity existed (*I*^2^ < 50%). We used the random-effects model when the *I*^2^ value was ≥50%. If high heterogeneity existed, a subgroup analysis of the cancer characteristics was conducted to determine possible causes. Differences between the subgroups were calculated depending on the Cochrane Handbook for Systematic Reviews of Interventions. Sensitivity analysis was conducted to validate the credibility of outcomes in our meta-analysis by assessing potential publication bias with visual inspection of the funnel plots.

## 3 Results

### 3.1 Search result and study characteristics

**Figure 1:**
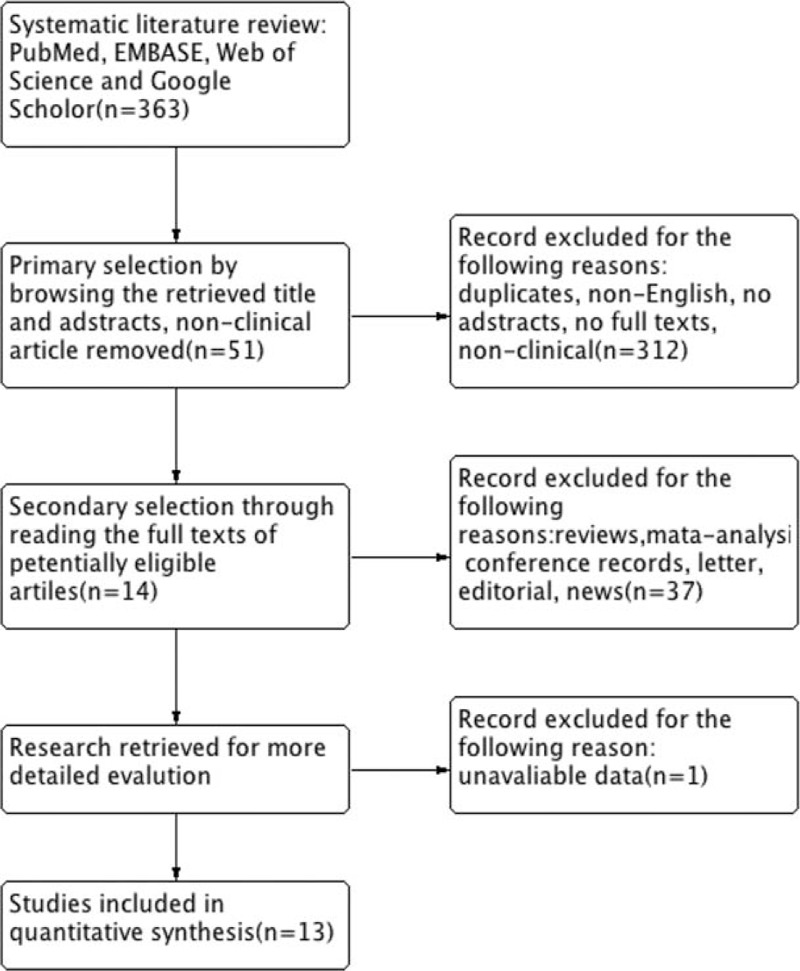
Flow diagram of the study selection process.

A total of 13 studies published between 2004 and 2015 with totally 2205 patients were eligible for the meta-analysis (Fig. 1). The main characteristics of the studies are reported in Table 3. Among all 13 study cohorts, there were 9 evaluated OC,^[17–25]^ 3 accessed EC,^[26–28]^ and 1 focused on US.^[29]^ On the other hand, studies were conducted in North of America (3), Europe (6), and Asia (4). For the outcome assessments, 20 datasets extracted from 13 studies were considerable. There were 8 OS, 4 DSS, 3 DFS, 2 recurrence-free survival (RFS), and 3 PFS. Since the definitions among DFS/RFS/PFS were not standardized in the majority of our analysis, we considered them equivalent and classified them as a group.

**Table 3:**
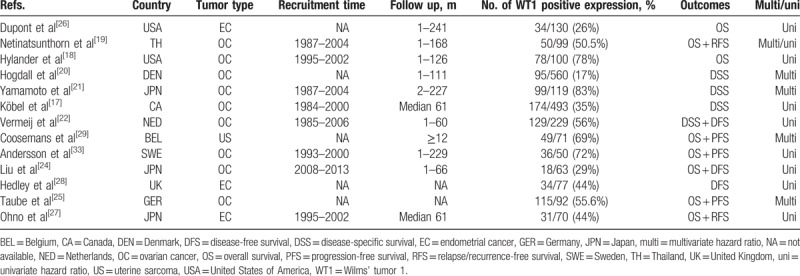
The main characteristics of the studies.

### 3.2 Main results of meta-analysis

We divided all outcomes into 3 groups including OS, DSS, and DFS/RFS/PFS. Then we presented the main results according to different groups.

**Figure 2:**
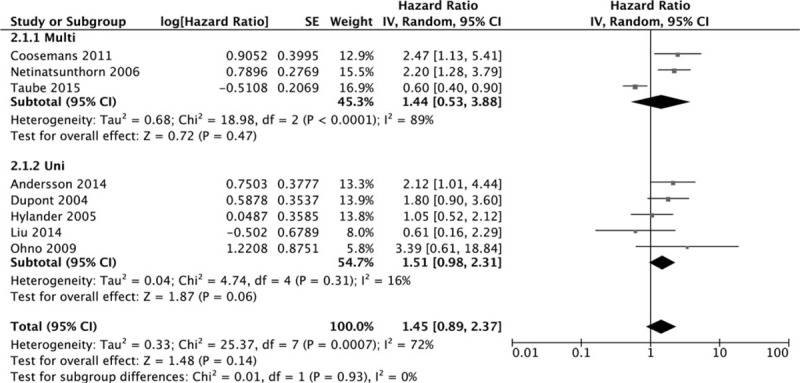
Forest plot of WT1 expression with OS.

**Figure 3:**
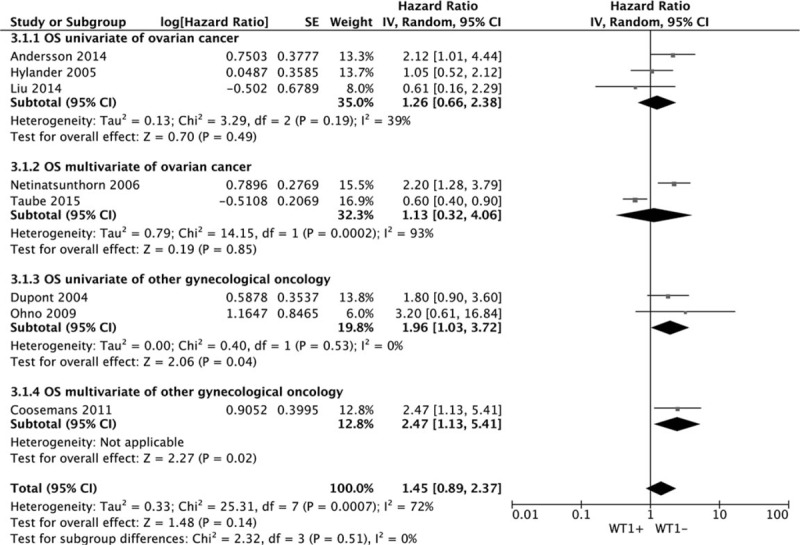
Forest plot describing the subgroup analysis WT1 expression with OS.

Eight datasets of OS showed that WT1 overexpression was insignificantly associated with OS. The combined HR estimate of OS was 1.45 (95% CI: 0.89–2.37). The insignificant association was showed in both univariate model (metaHR = 1.51, 95% CI = 0.98–2.31) and multivariate model (metaHR = 1.44, 95% CI = 0.53–3.88) (Fig. 2, Table 4). Subgroup analysis by cancer types revealed that WT1 overexpression did not have an unfavorable effect on OC in univariate model (metaHR = 1.26, 95% CI = 0.66–2.38) and multivariate model (metaHR = 1.13, 95% CI = 0.32–4.06). For other gynecological cancers except OC, the WT1's prognostic value was evaluated only in univariate model (metaHR = 1.96, 95% CI = 1.03–3.72). Because only 1 dataset indicate multivariate HR (Fig. 3). Subgroup analyses also did not show any significant associations, except for studies with sample size < 100 (metaHR = 2.00, 95% CI = 1.21–3.32) (Table 4).

**Table 4:**
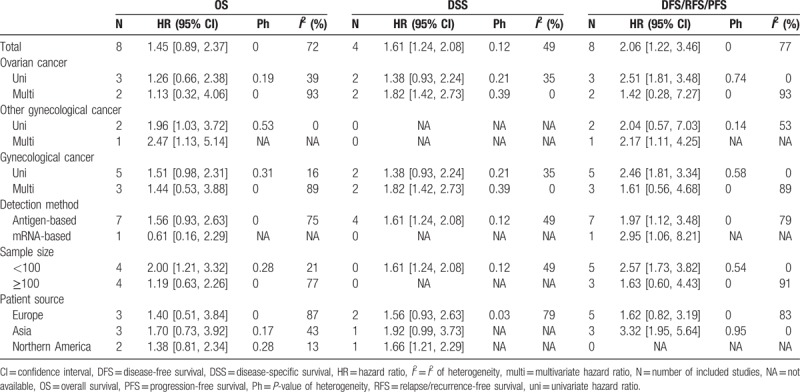
Results of this meta-analysis with different classifications.

**Figure 4:**
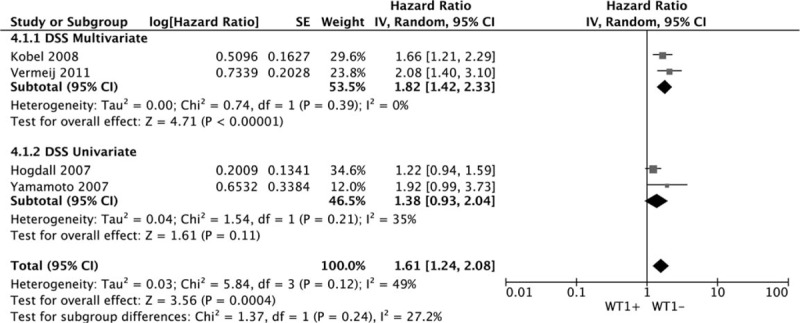
Forest plot of WT1 expression with DSS.

Four datasets of DSS showed that WT1 overexpression was significantly associated with DSS. The combined HR estimate of DSS was 1.61 (95% CI: 1.24–2.08) Fig. 4. All the datasets were reveled from OC using antigen-based method with a sample size < 100. The association was held only in multivariate model (metaHR = 1.82, 95% CI = 1.42–2.73), but not in univariate model (metaHR = 1.38, 95% CI = 0.93–2.24). The insignificant association was showed in both univariate model (metaHR = 1.51, 95% CI = 0.98–2.31) and multivariate model (metaHR = 1.44, 95% CI = 0.53–3.88) (Table 4).

**Figure 5:**
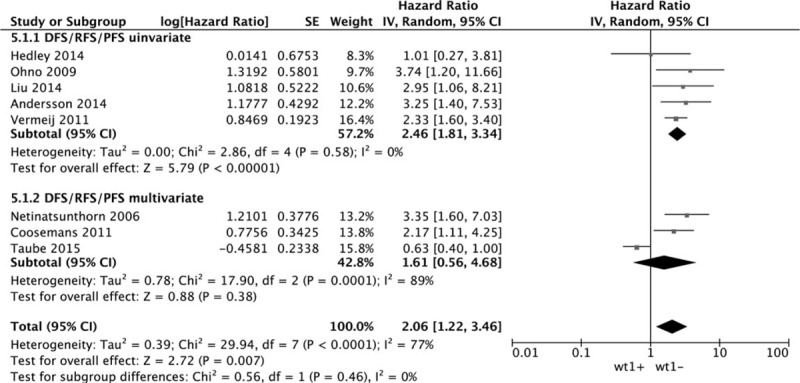
Forest plot of WT1 expression with DFS/RFS/PFS.

**Figure 6:**
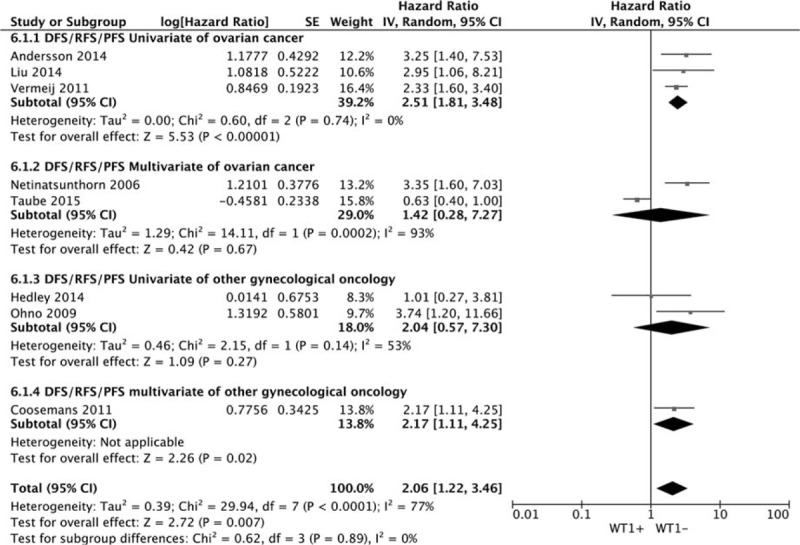
Foest plot describing the subgroup analysis WT1 expression with DFS/RFS/PFS.

Eight datasets of DFS/RFS/PFS showed that WT1 overexpression was significantly associated with DFS/RFS/PFS. The combined HR estimate of DFS/RFS/PFS was 2.06 (95% CI: 1.22–3.46). The association held only in univariate model (metaHR = 2.46, 95% CI = 1.81–3.34), but not in multivariate model (metaHR = 1.61, 95% CI = 0.56–4.68) Fig. 5. Subgroup analysis by cancer types revealed that WT1 overexpression had an unfavorable effect on OC in univariate model (metaHR = 2.51, 95% CI = 1.81–3.48). For other gynecological cancers except OC, the WT1 overexpression had an unfavorable effect in multivariate model (metaHR = 2.17, 95% CI = 1.11–4.25) Fig. 6. Subgroup analyses also did not show any significant associations, except for studies with a sample size < 100 (metaHR = 2.57, 95% CI = 1.73–3.82), with using antigen-based method (metaHR = 1.97, 95% CI = 1.12–3.48), and in Asia (metaHR = 3.32, 95% CI = 1.95–5.64) (Table 4).

### 3.3 Publication bias

**Figure 7:**
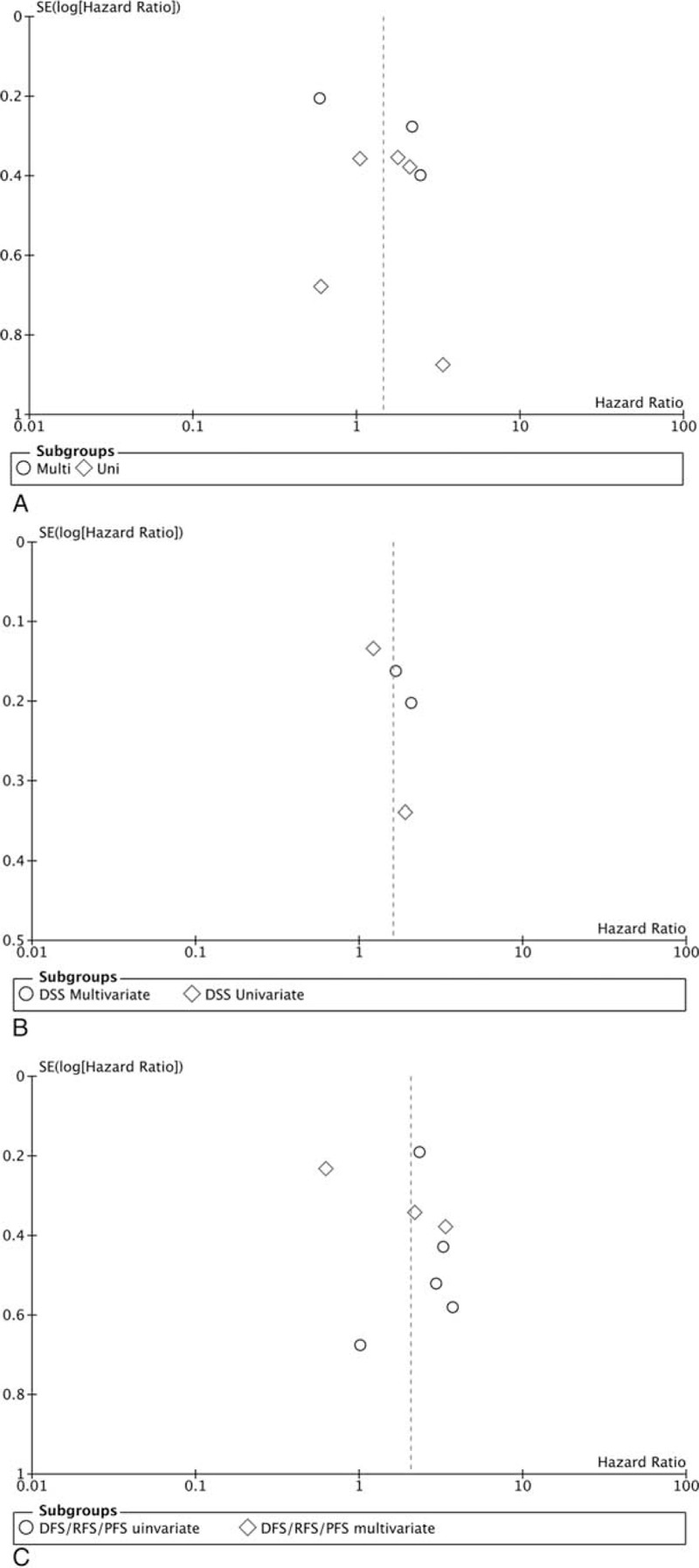
Funnel plots (7A for OS, 7B for DSS, 7C for DFS/RFS/PFS).

In this meta-analysis, Begg's funnel plot was used to evaluate the publication bias. The shape of the funnel plots did not show evidence of obvious asymmetry for OS (Fig. 7A), DSS (Fig. 7B), and DFS/RFS/PFS (Fig. 7C). The results above suggested the publication bias was not evident.

## 4 Discussion

WT1 was firstly discovered and regarded as a tumor suppressor gene in Wilms’ tumor^[17]^ and then it was also discovered as a suppressor in clear cell renal cell carcinoma (ccRCC).^[30]^ However, recent studies revealed that WT1 was expressed in a number of other tumors such as acute leukemia, breast cancer, brain tumors, and other tumors,^[31–35]^ in which WT1 might serve as an oncogenic role.^[4]^ Moreover, it was reported that WT1 could also promote invasion, migration and metastasis,^[36–38]^ facilitate angiogenesis^[39,40]^ and might be a promising target for immunotherapeutic treatment.^[22,41,42]^ It has been shown that the biological function of WT1 can be influenced by other interactive proteins like p53 and par-4.^[43,44]^ Qi et al^[45]^ reporting that the expression of WT1 showed a significant association with poor OS and DFS/RFS/PFS, and a borderline association with worse DSS, in patients with gynecological cancer. Moreover, they drawn subgroup analyses to detective the correlations of WT1 overexpression with OC. It shown that WT1 expression can only predict poor outcomes in univariate model, but not in multivariate model. In this meta-analysis, we find that the WT1 overexpression is contribute to poor outcome in DSS (metaHR = 1.61, 95% CI = 1.24–2.08), especially in univariate model (metaHR = 1.82, 95% CI = 1.42–2.08), which is accordance with Martin's, Köbel's,^[17]^ and Vermeij's^[22]^ study. In addition, poor outcome in DFS/RFS/PFS (metaHR = 2.06, 95% CI = 1.22–3.46) especially in univariate model (metaHR = 2.46, 95% CI = 1.81–3.34), is the same as Ohno's,^[27]^ Vermeij's,^[22]^ Andersson's,^[23]^ Liu's^[24]^ study but is opposite to Hedley's^[28]^ study. On the other hand, WT1 expression has a borderline association with worse OS (metaHR = 1.51, 95% CI = 0.98–2.31) in univariate model. From our point of view, in univariate analysis WT1 can becomes an independent unfavorable predictor for DSS, DFS/RFS/PFS and it can also predict OS in gynecological cancers. OC is mostly reported among these cancer types, so only taking OC into account can we find that the expression of WT1 is associated with unfavorable DSS (metaHR = 1.82, 95% CI = 1.42–2.33) and DFS/RFS/PFS (metaHR = 2.51, 95% CI = 1.81–3.48) in univariate model. Therefore, the WT1 expression can predict the poor prognostic for OC in univariate model. We suggest that the overexpression of WT1 may predict the prognostic and progression for these patients.

From some studies we find that overexpression of WT1 in high stage OC and US has significant poor outcome in OS, DSS, and DFS/RFS/PFS. It suggests that WT1 may be used as predictor to evaluate the prognosis of patients with high stage of gynecological cancer. WT1 was once used as a marker for serous tumor,^[46]^ Köbel's^[17]^ study showed that WT1 might be a significant prognostic factor in high-grade serous OC. In our meta-analysis, we find that serious OC with overexpression of WT1 can lead to unfavorable outcomes of DSS (metaHR = 1.85, 95% CI = 1.21–2.82), but not in OS (metaHR = 1.02, 95% CI = 0.35–2.95) and DFS/RFS/PFS (metaHR = 1.29, 95% CI = 0.39–4.21).

Several limitations should be considered when interpreting the findings of our meta-analysis. Differences among dilution solubility, antibodies, and cutoff values influence the assessment of WT1 overexpression. A large multicenter clinical study using consistent antibodies and cutoff values is needed to validate our results. What's more, we combined DFS/RFS/PFS as a group. Although definitions among DFS/RFS/PFS are not standardized in the majority of our analysis but we consider them equivalent, differences among them still existing and the combination can lead a bias. Language bias may exist in our meta-analysis because the search strategy was limited to English. Some studies did not report HR and 95% CI directly. Data extracted by using Tierney's methods may introduce bias to the original data.

In summary, this meta-analysis indicates that WT1 maybe a potential marker to predict the prognosis and progression for patients with gynecological cancer. However, more studies are needed to confirm these findings.

## Author contributions

Conception/design: Jingjing Lu, Yang Gu, Lanling Wen. Provision of study materials: Jingjing Lu. Collection and/or extract data: Yang Gu, Jingjing Lu, Qing Li, Huanxin Zhong; Data analysis and statistical guidance: Jingjing Lu, Xiaoxue Wang, Zhenxia Zheng, Wenfeng Hu. Final approval of the manuscript: Lanling Wen.

**Conceptualization:** Jingjing Lu, Lanling Wen, Xiaoxue Wang.

**Data curation:** Jingjing Lu, Yang Gu, Qing Li.

**Formal analysis:** Jingjing Lu, Yang Gu, Huanxin Zhong.

**Funding acquisition:** Zhenxia Zheng.

**Project administration:** Jingjing Lu, Lanling Wen, Wenfeng Hu.

**Resources:** Lanling Wen.

**Software:** Yang Gu.

**Validation:** Xiaoxue Wang.

**Visualization:** Yang Gu.

**Writing – original draft:** Jingjing Lu, Yang Gu.

**Writing – review & editing:** Jingjing Lu, Yang Gu, Lanling Wen.
